# Tiludronate treatment improves structural changes and symptoms of osteoarthritis in the canine anterior cruciate ligament model

**DOI:** 10.1186/ar3373

**Published:** 2011-06-21

**Authors:** Maxim Moreau, Pascale Rialland, Jean-Pierre Pelletier, Johanne Martel-Pelletier, Daniel Lajeunesse, Christielle Boileau, Judith Caron, Diane Frank, Bertrand Lussier, Jerome RE del Castillo, Guy Beauchamp, Dominique Gauvin, Thierry Bertaim, Dominique Thibaud, Eric Troncy

**Affiliations:** 1 Research Group in Animal Pharmacology of Quebec (GREPAQ) - Department of Veterinary Biomedicine, Faculty of Veterinary Medicine - Université de Montréal, 1500 des vétérinaires St., St.-Hyacinthe, QC J2S 7C6, Canada; 2 Osteoarthritis Research Unit, University of Montreal Hospital Research Centre (CRCHUM), Notre-Dame Hospital, 1560 Sherbrooke St. East, Montreal, QC H2L 4M1, Canada; 3 Clinical Exploration, CEVA Santé Animale, 10 av. de la Ballastière, Libourne, F-33500, France; 4 Development and Pharmaceutical Regulatory Affairs, CEVA Animal Health USA LLC, 8735 Rosehill Road, Lenexa, KS 66215, USA

## Abstract

**Introduction:**

The aim of this prospective, randomized, controlled, double-blind study was to evaluate the effects of tiludronate (TLN), a bisphosphonate, on structural, biochemical and molecular changes and function in an experimental dog model of osteoarthritis (OA).

**Methods:**

Baseline values were established the week preceding surgical transection of the right cranial/anterior cruciate ligament, with eight dogs serving as OA placebo controls and eight others receiving four TLN injections (2 mg/kg subcutaneously) at two-week intervals starting the day of surgery for eight weeks. At baseline, Week 4 and Week 8, the functional outcome was evaluated using kinetic gait analysis, telemetered locomotor actimetry and video-automated behaviour capture. Pain impairment was assessed using a composite numerical rating scale (NRS), a visual analog scale, and electrodermal activity (EDA). At necropsy (Week 8), macroscopic and histomorphological analyses of synovium, cartilage and subchondral bone of the femoral condyles and tibial plateaus were assessed. Immunohistochemistry of cartilage (matrix metalloproteinase (MMP)-1, MMP-13, and a disintegrin and metalloproteinase domain with thrombospondin motifs (ADAMTS5)) and subchondral bone (cathepsin K) was performed. Synovial fluid was analyzed for inflammatory (PGE_2 _and nitrite/nitrate levels) biomarkers. Statistical analyses (mixed and generalized linear models) were performed with an α-threshold of 0.05.

**Results:**

A better functional outcome was observed in TLN dogs than OA placebo controls. Hence, TLN dogs had lower gait disability (*P *= 0.04 at Week 8) and NRS score (*P *= 0.03, group effect), and demonstrated behaviours of painless condition with the video-capture (*P *< 0.04). Dogs treated with TLN demonstrated a trend toward improved actimetry and less pain according to EDA. Macroscopically, both groups had similar level of morphometric lesions, TLN-treated dogs having less joint effusion (*P *= 0.01), reduced synovial fluid levels of PGE_2 _(*P *= 0.02), nitrites/nitrates (*P *= 0.01), lower synovitis score (*P *< 0.01) and a greater subchondral bone surface (*P *< 0.01). Immunohistochemical staining revealed lower levels in TLN-treated dogs of MMP-13 (*P *= 0.02), ADAMTS5 (*P *= 0.02) in cartilage and cathepsin K (*P *= 0.02) in subchondral bone.

**Conclusion:**

Tiludronate treatment demonstrated a positive effect on gait disability and joint symptoms. This is likely related to the positive influence of the treatment at improving some OA structural changes and reducing the synthesis of catabolic and inflammatory mediators.

## Introduction

Osteoarthritis (OA) is among the most common musculoskeletal conditions [1]. This disease leads to functional disability and a reduced quality of life [2]. The abnormal biomechanics are believed to be among the major risk factors of disease progression and joint tissue damage [3].

Subchondral bone turnover is a well-defined component of OA [4]. The interactive process between articular cartilage and subchondral bone is complex and not yet fully understood. Yet, as these tissues are intimately related components of the joint, treatment to limit excessive bone remodelling is believed to have a possible positive effect on the global evolution of OA structural changes. Indeed, bone anti-resorptive agents have been shown to limit the development of OA structural changes in a number of experimental models [5]. For instance, inhibition of bone remodelling by licofelone [6] and calcitonin [7] in the experimentally transected canine anterior cruciate ligament (ACL) model of OA was shown to reduce cartilage lesions. Similar evidence also emerged from the work done on oestrogen replacement therapy in ovariectomized monkeys [8].

Bisphosphonates (BPs) are a well-known class of molecules that contain two phosphonate groups attached to a single carbon atom, forming a "P-C-P" structure. The antiresorptive effects of these biochemical analogs of inorganic pyrophosphate have been demonstrated in skeletal diseases where excessive bone resorption is present [9]. The anion of tiludronic acid (tiludronate, TLN) is a non-nitrogen-containing BP that acts on bone through mechanisms that involve induction of osteoclast apoptosis and prevention of extracellular degradation and of pro-inflammatory cytotrafficking [10], leading to decreased mineralized matrix resorption. This drug is recommended for skeletal disorders characterized by an increased and abnormal bone remodelling, such as Paget's disease, and is currently the only BP approved in veterinary medicine to alleviate clinical signs of an OA condition in horses [11]. There is yet insufficient data to claim a potentially beneficial effect of TLN on the pathological changes encountered in OA.

A recent study demonstrated that pre-emptive chronic zoledronate (a nitrogenous BP) treatment increases bone mineral density, and is chondroprotective and analgesic in both chemical (mono-iodo-acetate, MIA) and surgical experimental models of painful joint degeneration in the rat [12]. The authors showed that osteoclast-mediated resorption of cartilage at the subchondral bone/cartilage interface is an early initiating event in the pathobiology of the MIA model as opposed to chondrocyte death and subsequent mechanical erosion of the articular surface. Pre-emptive zoledronate fully inhibited the subchondral bone/cartilage molecular cross-talk [4,13] and/or the BP could have had a direct analgesic effect. This provided further rationale to test the potency of TLN at improving functional disability and structural changes in the canine ACL model of OA.

While BPs [14,15] and other antiresorptive agents [5-7,16] have shown promise, mostly structural effects (inhibition of cartilage degeneration [12,14], prevention of osteophytes [14] and reduction in bone marker turnover [15]) in animal models of OA with pre-emptive treatment, clinical results in knee OA patients have been disappointing, for example, with risedronate [17]. In OA, sclerosis of the subchondral bone is preceded by its resorption in the early phase [4,13,18]. This bone remodelling has also been characterized as bone marrow lesions (BML) on magnetic resonance imaging (MRI), and could also be perceived as an adaptation to changes in the biomechanics (maintaining intramedullary homeostasis [19]) or in an attempt to repair microdamages [18]. Therefore, bone remodelling has been associated with redistribution of mechanical stress [19], and the hypothesis has been advanced that to counteract it could prevent the repair of naturally occurring bone microdamage, thus increasing the susceptibility to crack initiation [20]. Moreover, in the ACL transection canine OA model, an experimental BP was demonstrated to be effective at reducing the turnover of cancellous subchondral bone, but ineffective at preventing osteophyte formation or pathologic changes of OA in the overlying cartilage [21]. Rather, a decrease in proteoglycan synthesis was observed, suggestive of impairment in the hypertrophic repair process. In contrast, chondroprotection was denoted in cruciate-deficient rats under BP treatment [12,14] in parallel to a decrease in the expression of degradative enzymes [14] as well as of biochemical markers of cartilage degradation in human beings [17]. Also, in these studies stating a limited efficacy for BP treatment [17,21], it has to be noted that one main limitation to inference was the relatively mild degree of OA in the control animals [21] as well as the absence of OA radiographic signs progression in placebo subjects [17].

ACL transection in dogs generates abnormal biomechanical forces and metabolic pathways that initiate structural changes on morphometry and histology [5], as well as on imaging [22,23], mimicking those seen in naturally occurring OA. This model is additionally acknowledged to induce significant chronic gait disability and functional impairment [24,25]. The canine ACL model of OA is valuable for assessing the evolution of functional outcomes in response to treatment [5]. In the present study, we hypothesized that the bone anti-resorptive action of TLN might curb the development of structural and functional joint lesions associated with ACL transection. We used a set of complementary tools to relate pain and functional outcomes in parallel to joint structural, biochemical and molecular changes. This allowed the evaluation of the effect of TLN on limb loading, pain/stress sensation, activity level and behaviours related to canine experimental OA conditions.

## Materials and methods

**Figure 1:**
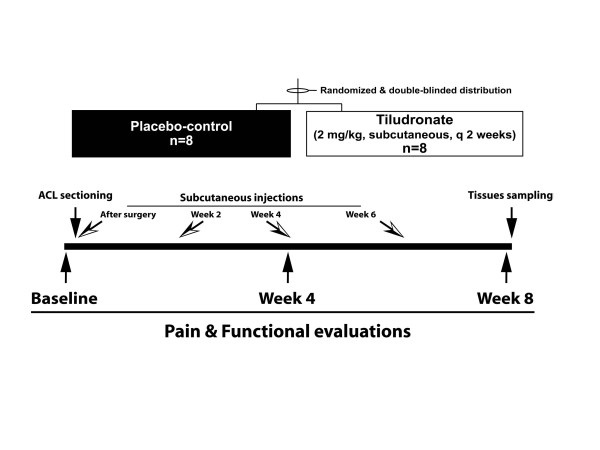
**Schematic representation of the study design**.

In this randomized, double-blind, placebo-controlled study with a parallel design (Figure 1), dogs were randomly allocated to two treatment groups of eight dogs each, stratified by body weight and gender. Investigators were blinded to group allocation, as well as treatment. The study protocol was approved by the institutional animal care and use committee (RECH-1268) and conducted in accordance with the Canadian Council on Animal Care guidelines.

### Animals

Sixteen adult crossbred dogs (aged two to three years), with an average (SD) body weight of 26 (3.3) kg were used in this study. They were individually housed in galvanized steel cages (1 m (width) × 1.75 m (length) × 2.4 m (height)) fitted with automatic waterers. Dogs were included in the study after complete physical and musculoskeletal evaluation by a veterinarian, and haematological and biochemical analyses. Food (approximately 450 g Hill's Pet Nutrition Science Diet Canine Adult Original mixed with Harlan Teklab Global 27% Protein Dog Diet) was given once daily and removed overnight. Tap water (purified by filtration) was provided to the animals *ad libitum*.

### Surgical transection of the anterior cruciate ligament (ACL)

After evaluation of baseline pain and functional outcome levels, all anaesthetized dogs were subjected to ACL transection of the right knee as previously described [26]. Under pre-emptive (transdermal fentanyl 50 or 75 μg/h, Duragesic^®^; Janssen Ortho, Markham, ON, Canada) and multimodal (intra-articular block combined with opioid administration) analgesia, the tibial edge was located with the thumb and index finger, followed by a medial sagittal skin incision (30 mm). The subcutaneous tissues were dissected, and a medial arthrotomy was performed, distal to the patella and parallel to the patellar ligament. A retractor was inserted to view the ACL to be sectioned, the completeness of which was verified by obtaining a large drawer motion in both flexion and extension. The capsule and the retinaculum were sutured in a simple continuous pattern. Bupivacaine (Marcaïne^® ^0.5%; Hospira, St-Laurent, QC, Canada) was injected (5 to 8 mL) in the capsule as an intra-articular block. Finally, the subcutaneous tissues were sutured, followed by intra-dermal and skin sutures.

### Treatment

One group was treated with 2 mg/kg of disodium TLN dissolved in a mannitol solution (CEVA Santé Animale, Libourne, France). The OA control group received only the vehicle solution (CEVA Santé Animale). Both treatments (0.2 mL/kg) were injected subcutaneously (SC), starting on the day of ACL transection, and repeated every two weeks up to the end of the follow-up (total of four administrations). The dose level of TLN was selected by the Sponsor based on preliminary studies in rats [27] and using an allometric scale-up to the weight of dogs.

### Pain and functional evaluations

#### Gait analysis

In dogs, the use of a pressure-sensing walkway device acquires limb loading and is defined as a quantitative measurement of gait function [25]. Gait analysis was performed at baseline, Week 4 and Week 8 using the podobarometric recording device (Walkway^® ^System; Tekscan Inc, Boston, MA, USA) [25,28]. For the right (ACL-deficient) hind limb, the peak vertical force (PVF) was acquired at a trotting gait velocity ranging from 1.9 to 2.2 meters/second. Velocity and acceleration (± 0.5 meter per second^2^) was ensured using a set of three photoelectric cells specially designed for this podobarometric device (LACIME; École de Technologie Supérieure, Montréal, QC, Canada). The gait acquisition window was three seconds with a sampling rate set at 44 hertz, producing a total of 132 frames. Raw PVF (Kg) data from the first five valid trials were obtained for each dog and later used for statistical purposes using body weight as a covariate [25]. Data were expressed as percentage of body weight (%BW).

#### Pain scoring systems

The lameness and pain of treated and control OA dogs were evaluated using previously developed scoring systems, and included a visual analog scale (VAS) [29] and a composite numerical rating scale (NRS) [30]. The pain scores were obtained at baseline, Week 4 and Week 8 by the same technician [29] with a 100 mm VAS scale, coding from 0 ("no pain") to 100 ("pain intensity could not be worse"). The composite NRS, which was scored by the same veterinarian throughout the study, includes the following seven criteria: Global assessment (score 0 to 4); Evaluation of lameness while the dog is standing up (score 0 to 4), walking (score 0 to 4) and trotting (score 0 to 4); Willingness to hold up contralateral limb (score 0 to 4); Evaluation of response to palpation (score 0 to 4); Evaluation of response to flexion and extension (score 0 to 4). Inter- and intra-observer reliability of both VAS and NRS were tested and found to be highly satisfying (Spearman correlation, rho >0.72, *P *< 0.001).

#### Electrodermal activity (EDA)

Changes in skin conductance response (EDA) resulting from sympathetic neuronal activity [31] has recently been validated in the canine ACL model of OA as a measurement of stress or pain that is strongly associated with functional outcomes [30]. The EDA was recorded at baseline, Week 4 and Week 8 using a Pain Gauge^® ^(PHIS, Inc., Dublin, OH, USA) system, which grades the signal intensity on a scale of 0 to 10, with 10 being the most painful. The device was placed for two seconds on the right palmar paw (dry and non-clipped).

#### Video-automated behaviour analysis

The computer-assisted behavioural analysis (The Observer v5.0.31; Noldus Information Technology, Inc., Leesburg, VA, USA) allowed the assessment of behavioural changes suggestive of a pain-related condition [32]. The capture of behavioural changes in terms of body positions and motor activities allows a non-invasive monitoring of pain-related functional disability and discomfort. Recording was performed in the outdoor runs where the dogs exercised for two consecutive hours, at baseline and at Week 8. The resulting ethogram included the following eight classes of behaviour: location in the run, body position, facial expression, vocalization, tail position, self-care, motor activity and dog interaction. The analysis of behaviour occurrence was done following the manufacturer's recommendations.

#### Telemetered locomotor actimetry

Acceleration-based monitoring of frequency, intensity, and duration of physical activities is a valid objective tool to monitor pain-related functional disability [30,33]. At baseline, Week 4 and Week 8, locomotor actimetry was monitored continuously for 24 hours with an electronic chip (Actical^®^; Bio-Lynx Scientific Equipment, Inc., Montreal, QC, Canada) that was placed inside a protective neck collar. During this time, all dogs followed the same daily routine to ensure consistency. The cumulative locomotor activity was recorded over two minutes, thus providing 720 measurements over 24 hours. The height of peaks for each recording was scaled in arbitrary units from 0 to ∞ to quantify the intensity of locomotor actimetry [30]. Comparison of actimetry with simultaneous video-automated recordings allowed the threshold to be set between active and inactive motions at 30 units. Data were then expressed as daily averaged total intensity (considering all counts, DATI), and daily averaged active intensity (considering only counts higher than 30 in intensity as active counts, DAAI).

### Macroscopic grading

At the end of the study, the dogs were euthanized under sedation with barbiturate overdose. The right knee of each dog was placed on crushed ice and dissected for quantification of gross morphological changes. Two independent observers who were blinded to treatment group allocation graded the findings with a consensual value [25,26,34].

#### Cartilage

Macroscopic lesion areas at the cartilage surface on the femoral condyles and tibial plateaus were measured (in mm^2^) with an electronic digital calliper (Digimatic Caliper model No. 2071M; Mitutoyo Corporation, Kawasaki, Japan). The depth of erosion was graded with scores ranging between 0 (a normal surface) and 4 (erosion extending to the subchondral bone).

#### Osteophytes

When present, the degree of osteophyte formation was quantified by measuring the maximal width (mm) of the spurs on the medial and lateral femoral condyles as previously described [26]. For statistical purposes, data were evaluated separately for lateral and medial osteophytes and also summated for the entire condyles.

### Histological grading of cartilage and synovial membrane

#### Cartilage

Full thickness cartilage sections were removed from the weight-bearing lesional areas of the femoral condyles and tibial plateaus allowing standardization of sampling [25]. Histological evaluation was performed on sagittal sections of cartilage from each femoral condyle and tibial plateau specimen [25,26,34]. After dissection, specimens were fixed in 4% buffered formalin and embedded in paraffin. Serial sections (5 μm) were stained with haematoxylin/Fast green and Safranin-O. The severity of cartilage pathology was graded by two independent observers using the OsteoArthritis Research Society International histopathology scoring system [35]. For statistical purposes, data from both observers were considered for the lateral and medial part of the condyles and plateaus as well as for the entire joint.

#### Synovial membrane

Synovial membrane was removed and processed as described above, but stained with haematoxylin/eosin. Two independent observers evaluated two specimens. The severity of synovitis was graded on a scale of 0 to 10, including four histological criteria as previously described [34]: synovial cell hyperplasia (scale 0 to 2), villous hyperplasia (0 to 3) and mononuclear (0 to 4) and polymorphonuclear (0 to 1) cell infiltration; 0 indicates normal structure. For statistical purposes, data from both observers and both specimens were considered.

### Analysis of synovial fluid

At euthanasia, samples of synovial fluid were collected, measured, and then centrifuged and frozen (-80°C).

#### Prostaglandin (PG) E_2 _assay

The amount of PGE_2 _(ng/knee) was determined using a commercially available Enzyme ImmunoAssay (Cayman Chemicals, Ann Arbor, MI, USA) according to the manufacturer's instructions, the limit of detection being 15 pg/mL. The concentration measurements were done in duplicate and the values averaged.

#### Nitrites and nitrates (NOx) assay

Nitrite and nitrate levels (nmol/knee) were determined by chemiluminescence [36] using a NO Analyzer (280i^®^, Sievers Instruments, Boulder, CO, USA), according to the manufacturer's instructions. Briefly, 0.025 mL of the supernatant was injected into the microreaction purge vessel of the analyzer. The purge vessel contained 5 mL of vanadium solution heated at 95°C. The instrument measures NOx on a gas-phase chemiluminescent reaction between NO and ozone. Each sample was analyzed in duplicate and values averaged.

### Immunohistochemistry

Full thickness specimens from the tibial plateaus and femoral condyles were processed for immunohistochemical analysis, as previously described [6,26,34]. After the slides were incubated with a blocking serum (Vectastain ABC kit; Vector Laboratories, Inc., Burlingame, CA, USA) for 60 minutes, they were blotted and then overlaid with the primary antibody against the following: matrix metalloproteinase (MMP)-1 (1/40 dilution, mouse monoclonal; Calbiochem ref. #444209; EMD Biosciences, Darmstadt, Germany), MMP-13 (1/6, goat polyclonal antibody; R&D Systems, Minneapolis, MN, USA), a disintegrin and metalloproteinase domain with thrombospondin motifs (ADAMTS)5 (1/50, rabbit polyclonal; Cedarlane ref. #CL1-ADAMTS5; Hornby, ON, Canada), and cathepsin K (1/200, goat polyclonal; Santa Cruz Biotechnology ref. #sc-6506; Santa Cruz, CA, USA) for 18 hours at 4°C in a humidified chamber.

Each slide was stained using the avidin-biotin complex method (Vectastain ABC kit), and incubated in the presence of the biotin-conjugated secondary antibody for 45 minutes at room temperature followed by the addition of the avidin-biotin-peroxidase complex for 45 minutes. The slides were counterstained with eosin. Determination of the staining specificity as well as the immunohistochemistry analysis (three fields from each specimen examined) was done as previously described [6,26,34]. Each section was examined under a light microscope (Leitz Orthoplan; Leica, Inc., St. Laurent, QC, Canada) and photographed using a CoolSNAP of Photometrics camera (Roper Scientific, Rochester, NY, USA). The results are expressed as the percentage of cells staining positive for the antigen (MMP-1, -13 and ADAMTS5) in the upper zone of cartilage with the maximum value being 100%. Similarly, on decalcified specimens (see the Histomorphometry section), the number of cathepsin K positive cells was quantified in the subchondral bone, as previously described [6]. For statistical purposes, data from all specimens (tibial plateaus and femoral condyles) and all fields were considered. The data presented are the average of the three fields.

### Histomorphometry

Specimens of full-thickness sections of the articular cartilage including the subchondral bone from the lesional area of the medial tibial plateau of all dogs were placed in 10% (vol/vol) formalin before being decalcified with 10% (vol/vol) formic acid in formalin for 12 hours and embedded in paraffin, as previously described [6].

#### Subchondral bone

Sections (5 μm) of each specimen were subjected to Fast green/Safranin-O staining. A Leitz Diaplan DMLS^® ^microscope (Leica Microsystems, Wetzlar, Germany) connected to a personal computer (Pentium IV, using Image J software, v1.27; NIH, Bethesda, MD, USA and OSTEO II Image Analysis software; Bioquant, Nashville, TN, USA) was used to conduct the subchondral bone histomorphometry, which was performed as previously described [6,26]. Measurement of the bone surface (mm^2^), trabecular thickness (μm) and trabecular separation (mm) was done according to standard conventions [6].

#### Calcified cartilage

The calcified cartilage histomorphometry was done for each specimen, as previously described [6]. The surface (mm^2^) of the calcified cartilage was calculated using the computerized program.

### Statistical analysis

Linear mixed models for repeated measures were used to evaluate the effect of Time, Group and Time per Group interaction for PVF, EDA and actimetry recording using compound symmetry covariance structures. Trials (PVF) and dogs were random effects nested in treatment groups. At each time point, a group's least squares means were compared with appropriate Tukey or Bonferroni adjustments. To evaluate the effect of Time, Group and Time per Group interaction on VAS and summated NRS, repeated-measures generalized linear models with generalized estimating equations were used, where data were assumed to distribute under the Log-Gamma, and the overdispersed Poisson probability functions, respectively. For the latter variable, the variance scale factor was estimated by Pearson's chi-square/Degree of freedom. Best working matrix was determined to be first-order autoregressive following the strategy proposed by Littell *et al. *[37].

For the video-automated behaviour analysis, the occurrence of each specific event was cumulated. Frequencies were then compared between TLN and placebo control groups using a negative binomial regression model with baseline occurrence as covariate. Values were expressed as changes in the frequency of a given behaviour according to the tested group.

Synovial fluid volume, levels of inflammatory factors, cellular ratios and structure measurements were tested between groups using linear mixed models. Where appropriate, specimen or fields were used as random effects nested in treatment groups. Data presented as scores or counts were tested using a generalized linear model under the logistic polytomous distribution function using the proportional odds assumption, or the over-dispersed Poisson probability functions when scores were summated. Where appropriate, specimen or observers were used as random effects nested in treatment groups. Alpha threshold for significance was set at 5%. Data are presented as mean (SD). Statistical analyses were done using SPSS Statistics software v17.0 (SPSS Inc, Chicago, IL, USA) and SAS software, v9.1, (SAS Institute Inc, Cary, NC, USA).

## Results

### Pain and functional outcomes

**Figure 2:**
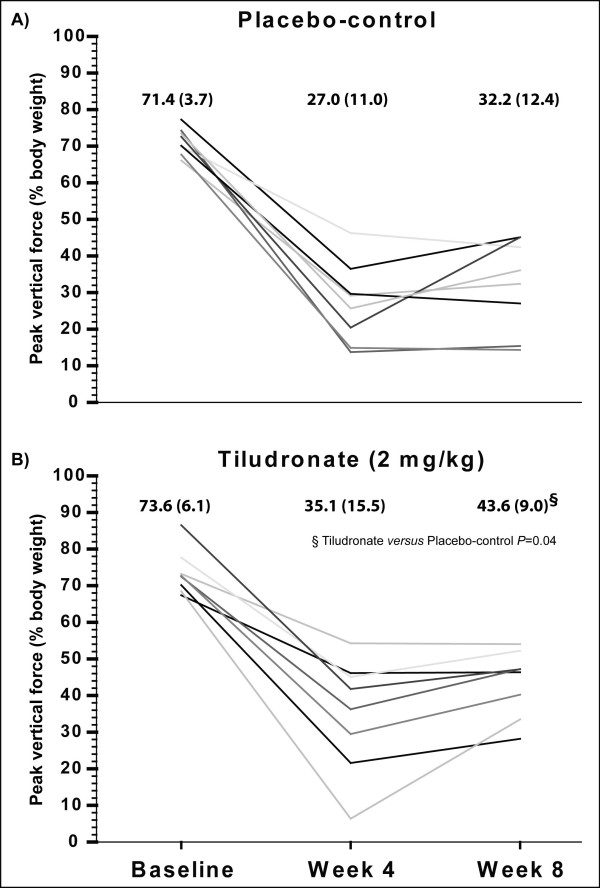
**Kinetic gait analysis**. Peak vertical force (mean (standard deviation)) recorded before (baseline) and four and eight weeks after anterior cruciate ligament transection in dogs. Line plots representation includes respective values for **A**) placebo-control and **B**) tiludronate treated dogs. There was a significant time effect (*P *< 0.01), a group effect (*P *= 0.05) and a time per group interaction (*P *< 0.01) with PVF value reaching 35% higher in tiludronate than in placebo-control at Week 8 (*P *= 0.04).

The temporal evolution of PVF recording implied severe gait disability denoted by decreasing values over time (*P *< 0.01) (Figure 2, Table 1). Treatment interacted with the temporal recording of PVF (*P *< 0.01). The TLN treatment over an eight-week duration provided reduction in the limb impairment compared to the placebo control over time (*P *= 0.05), reaching PVF values 35% higher at Week 8 (*P *= 0.04).

**Table 1:** Pain and functional outcomes before and after anterior cruciate ligament transection in dogs

	Time			
Evaluation methods/Groups	Baseline	Week 4	Week 8	*P-*values
Function - kinetic gait analysis (%BW)				
**Placebo-control**	71.4 (3.7)	27 (11.0)	32.2 (12.4)	*<0.01
**Tiludronate**	73.6 (6.1)	35.1 (15.5)	43.6 (9.0)	§ = 0.05 ¶ <0.01
Pain - Visual analog scale (VAS, measurement)				
**Placebo-control**	0.0 (0.0)	37.6 (14.3)	26.8 (11.1)	*<0.01
**Tiludronate**	0.0 (0.0)	26.9 (18.9)	15.6 (9.2)	
Pain - Numerical rating scale (NRS, score)				
**Placebo-control**	0.0 (0.0)	19.4 (4.3)	18.3 (3.7)	
**Tiludronate**	0.0 (0.0)	15.5 (5.4)	15.0 (3.4)	§ = 0.03
Pain - Electrodermal activity (EDA, reading)				
**Placebo-control**	4.5 (2.5)	6.4 (2.5)	5.3 (2.4)	
**Tiludronate**	4.2 (2.7)	3.6 (2.5)	3.9 (2.4)	
Function - Telemetered actimetry recording (count)				
Daily averaged total intensity (DATI, no unit)				
**Placebo-control**	97.9 (41.4)	79.1 (22.7)	85.7 (35.8)	
**Tiludronate**	82.3 (25.4)	104.5 (44.6)	91.2 (33.1)	¶ = 0.04
Daily averaged active intensity (DAAI, no unit)				
**Placebo-control**	390.9 (101.3)	360.6 (73.9)	379.1 (127.1)	
**Tiludronate**	390.2 (82.9)	502.6 (145.7)	443.1 (117.1)	¶ = 0.04

Tiludronate was injected subcutaneously at 2 mg/kg, starting immediately on the day of ACL transection and repeated every two weeks for an eight-week follow-up. Placebo-control dogs received mannitol injection in a similar fashion.

Data presented are mean (SD).

Statistically significant Time effect (*), Group effect (§) and Time per Group interaction (¶)

Assessment provided by VAS and NRS (Table 1) echoed the temporal evolution observed with gait analysis (PVF): After surgery, both methods detected a worsening of the dogs' condition. Assessment provided by VAS revealed an improvement from Week 4 to Week 8 (*P *< 0.01) in both groups, without the presence of an interaction of treatment on VAS recording. Of note, TLN dogs tended to have lower VAS grades than controls (*P *= 0.06). With respect to NRS, groups were not significantly improved from Week 4 to Week 8 and no interaction was denoted. However, there was a significant group effect (*P *= 0.03) on the overall NRS recorded post surgery.

According to EDA values (Table 1), the level of pain/stress sensation recorded after surgery did not change over time in the TLN-treated dog group, whereas in the placebo control group, the maximal increase in EDA was noted at Week 4. As a result, the interaction of treatment on the temporal evolution of EDA recording demonstrated a trend (*P *= 0.07) without denoting group effect.

According to the video-automated analysis, there was a significant increase in the relative frequencies of two major body position behaviours suggestive of comfort for TLN dogs: Full weight bearing while standing with head down increased by a factor of 2.92 (*P *= 0.03); Full weight bearing while standing and looking around increased by a factor of 7.7 (*P *= 0.03). Similarly, two motor activities (gait) were also more frequently observed in this group: Normal walking, factor of 5.22 (*P *= 0.01); and normal trotting, factor of 6.34 (*P *= 0.01).

The telemetered recording of DATI and DAAI denoted higher movement in TLN dogs following surgery (Table 1). Conversely, placebo control dogs were less active than at baseline. This divergence led to the discernment of an interaction (*P *= 0.04, DATI and DAAI) of treatment on the temporal evolution of telemetered actimetry, which resulted in higher DATI recording in TLN dogs when compared to placebo control at Week 4 (*P *= 0.05).

### Synovial fluid

The amount of joint effusion in the TLN-treated dogs (6.7 (2.8) mL) was significantly less (*P *= 0.01) than that found in the placebo control dogs (15.0 (7.6) mL). The TLN-treated dogs also had lower levels of PGE_2 _(*P *= 0.02) (4.4 (3.6) ng/knee) and NOx (*P *= 0.01) (306.2 (267.1) nmol/knee) than those treated with placebo (11.7 (7.2) ng/knee, and 766.28 (379.0) nmol/knee, respectively).

### Cartilage

#### Macroscopy

The severity of macroscopic lesions (depth and lesion surface) on the femoral condyles and tibial plateaus of TLN-treated dogs was not different from that observed in placebo control dogs (data not shown). The size of the osteophytes was similar between groups, both on medial (TLN; 6.7 (2.6) mm, placebo control; 7.0 (1.9) mm) and lateral (TLN; 6.5 (1.4) mm, placebo control; 7.1 (2.1) mm) condyles.

#### Histology

Histological grading of cartilage lesions did not reveal significant difference between groups. The synovial membrane score of TLN dogs (total score of 6.8 (1.1)) was similar to the placebo control dogs (total score of 6.1 (1.1)). The subset analyses revealed that the synovial lining cell score was significantly (*P *< 0.01) less in TLN dogs (1.0 (0.0)) compared to placebo control (1.7 (0.6)).

### Immunohistochemistry

**Figure 3:**
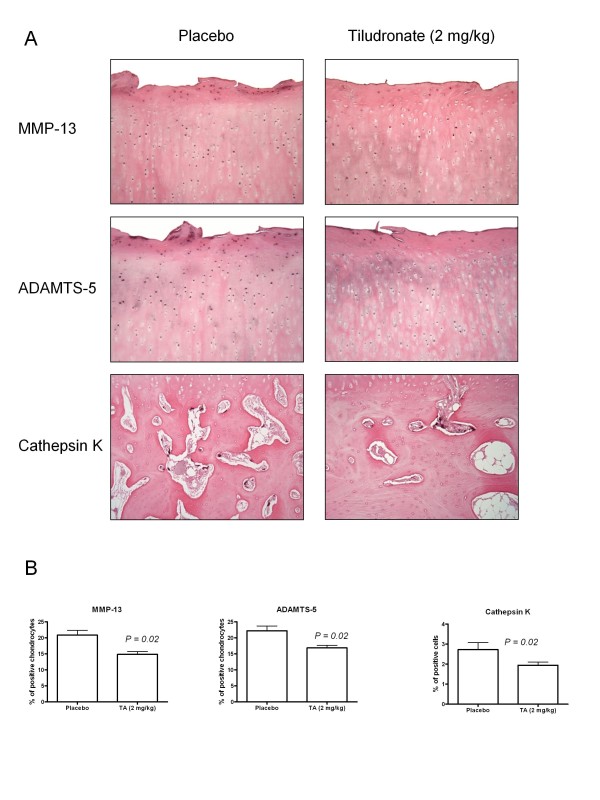
**Immunohistochemistry**. (**A**) Expression of matrix metalloproteinase 13 (MMP-13), ADAMTS5 and cathepsin K in representative sections of cartilage (MMP-13 and ADAMTS5) and subchondral bone (cathepsin K) from placebo-treated dogs with osteoarthritis (OA) and tiludronic acid (TA: 2 mg/kg/2 weeks)-treated dogs with OA. Positive cells are shown by dark brown staining (original magnification ×100). (**B**) Levels of MMP-13, ADAMTS5 and cathepsin K as determined by immunostaining. The values are expressed as the mean ± SEM. *P*-values were calculated by using a generalized linear model under logistic polytomous distribution function using the proportional odds assumption.

The cartilage immunohistochemistry revealed a significant decrease in the percentage of cells staining positive in TLN-treated dogs compared to the placebo control (Figure 3) for MMP-13 (14.9 (2.5)% *vs. *20.9 (4.2)%; *P *= 0.02) and ADAMTS5 (16.9 (2.3)% *vs. *22.2 (4.2)%; *P *= 0.02). The level of MMP-1 was similar in both groups (data not shown). Immunohistochemical analysis of the calcified cartilage revealed a slight decrease in the level of MMP-13 in TLN-treated dogs (19.7 (5.9)%) compared to control dogs (22.4 (2.3)%, NS). In the subchondral bone, the cathepsin K expression was significantly lower in dogs that had received TLN compared to placebo (1.9 (0.6) *vs. *2.7 (0.8), *P *= 0.02) (Figure 3).

### Histomorphometry

**Figure 4:**
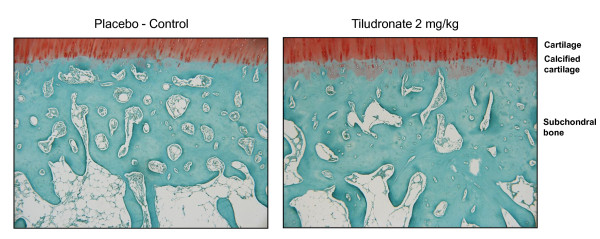
**Histomorphometry**. Representative histological sections of calcified cartilage and subchondral bone in osteoarthritic dogs that received either placebo (*n *= 8) or treatment with tiludronate (*n *= 8) 2 mg/kg/2 weeks. Specimens were selected from lesional areas of the tibial plateaus (Fast green/Safranin O staining, original magnification ×63).

The surface of the calcified cartilage demonstrated a trend (*P *= 0.07) to be greater in the TLN-treated dogs compared to the placebo dogs (Table 2, Figure 4). Dogs treated with TLN also had a significantly greater (*P *< 0.01) subchondral bone surface and smaller trabecular separation compared to control dogs. Trabecular thickness was similar in both treatment groups. The values for TLN-treated dogs were similar to those previously reported for normal dogs [6].

**Table 2:** Histomorphometry data eight weeks after anterior cruciate ligament transection in dogs

	**Calcified cartilage surface (10**^**-2 **^**mm**^**2**^**)**	**Subchondral bone surface (10**^**-2 **^**mm**^**2**^**)**	**Trabecular thickness (10**^**-2 **^**mm)**	**Trabecular separation (10**^**-3 **^**mm)**
**Placebo-control**	16.9 (3.9)	75.1 (9.3)	8.9 (2.3)	86.0 (52.6)
**Tiludronate**	19.8 (2.3)	86.2 (5.1)*	10.3 (2.0)	45.5 (15.1)*

Tiludronate was injected subcutaneously at 2 mg/kg, starting immediately on the day of ACL transection and repeated every two weeks for an eight-week follow-up. Osteoarthritic placebo-treated dogs received mannitol injection in a similar fashion.

Data presented are mean (SD). * Statistically significant (*P *< 0.01) compared to placebo-control.

## Discussion

Studies in the dog ACL model have provided insight into OA mechanisms and pathophysiological pathways that surround the evolution of the degenerative process. The model was proven to be most useful at the preclinical stage of drug development for testing the abilities of new therapeutic modalities to limit or halt the disease development/progression [5]. In the present study, TLN was demonstrated to have a positive effect both on some of the structural changes and on pain/function. More particularly, TLN was found to decrease the production of catabolic enzymes, bone resorption, and synovial inflammation. These were associated with improved locomotion, reduced lameness and gait disability, and improved joint pain perception. These findings are in line with the report on a rat model of OA [12] showing that the analgesic effect of the nitrogenous BP zoledronate is mediated by its anti-resorptive action. In the current study, TLN promoted better function despite the presence of cartilage lesions, which were of similar extent in OA and control dogs. This finding suggests that cartilage integrity in a weight-bearing joint is not compulsory for functional improvement. Furthermore, even when the knee joint was exposed to additional mechanical constrains related to an increase in limb support (that is, pain-relief), cartilage did not undergo excessive alteration and remained similar to control dogs.

This study demonstrated that TLN improves the functional disability following ACL transection, more specifically the limb impairment, allowing dogs to load their afflicted limb to a greater extent as demonstrated by the results of the PVF analysis. Dogs were also more active without showing evidence of severe lameness. The stress/pain sensation was also reduced by TLN treatment, as highlighted by the results of the EDA measurements, pain scoring and behaviours denoting changes in pain-related condition. Lameness improvement was found to be maximal at Week 8 (PVF, NRS and video-automated analysis of body position and motor activities), after dogs had received full TLN treatment. However, at the intermediate time-point (Week 4), the difference between TLN and placebo groups was greater than at Week 8 for both EDA and telemetered actimetry. The beneficial effect of TLN therapy on pain/function was likely related to a combination of effects including its anti-inflammatory effect shown by a reduction in synovial effusion size, synovitis score, and level of inflammatory mediators, as well as its effect on the initiation of bone remodelling [12].

Transduction of noxious signals occurs through high-threshold receptors responding to a variety of thermal, chemical and mechanical stimuli, and defined as polymodal nociceptors. At the level of damage-sensing neurons, release of protons and high concentrations of adenosine triphosphate (ATP) act, respectively, on transient receptor potential (TRP) and acid-sensing (ASIC) ion channels, and on ionotropic ligand-gated purinergic (P2X) receptors to activate nociceptors [38]. During the early stages of inflammation, mediators such as PGs and bradykinin change the sensitivity of receptors and reduce the activation threshold for these conducting ion channels, which is the basis for peripheral sensitization. This study provides interesting new findings on the ability of TLN to reduce the inflammatory changes in the OA synovium. Hence, TLN demonstrated a clear anti-inflammatory local effect while decreasing the joint effusion and synovitis (synovial lining cells). The analyses of synovial fluid confirmed the anti-inflammatory effects reflected by a decrease in the levels of PGE_2 _and NOx, two well-known inflammatory mediators [39]. Inflammatory markers such as PGE_2 _are known to be correlated with pain and functional disability in human OA [40] as well as in the canine ACL model of OA [41]. The high level of synovial fluid NOx found in this study is in line with our previous findings in this OA model, in which the inducible NOS was found to be increased [16,34]. It is likely that NO contributed to the disability and perception of pain [42]. Moreover, the decrease in NOx levels by TLN treatment may have contributed to the structural protective effect of the drug as previously demonstrated in this OA model [16]. Tiludronate, by inhibiting the ATP-dependent proton pumps located in the plasma membranes of the osteoclasts [43], can reduce the acidification of the bone matrix, which is the first step in the bone resorption process. In addition, TLN can disrupt adhesion of the osteoclast to the bone surface before bone resorption is initiated by modifying the phosphorylation of proteins of the cytoskeleton [44]. As well as decreasing pro-inflammatory cytotrafficking (cytokines and NO synthesis) [10], acidification and phosphorylation and, in consequence, activation of proteolytic enzymes, TLN can also inhibit activity of MMPs [45]. In the present study, TLN was found to decrease the expression of MMP-13 and ADAMTS5 in the cartilage and cathepsin K in the subchondral bone, which is in line with the findings of our previous study [6] and supports this hypothesis. The anti-inflammatory effects of TLN are interesting, as they add to the most important recognized biological effect of BPs, that is, the reduction in bone remodelling through the inhibition of osteoclastic activity. The pain and functional improvements observed under TLN therapy were present at Week 4 and maintained at Week 8. Therefore, it could be hypothesized that the actions of TLN on bone matrix and synovial inflammation would lead to a decrease in TRPV1, ASIC3, and P2X receptors activation, translating into diminished peripheral sensitization and subsequent benefits on pain sensation and mobility. Although BPs were developed for the treatment of pathologies associated with excessive bone resorption, several reports revealed that they were able to reduce the pain associated with different painful diseases. Direct analgesic effects of BPs have been largely acknowledged using reflex algesimetric tests in mice after acute administration [46]. Clodronate and pamidronate presented anti-nociceptive dose-responses, comparing favourably to aspirin and morphine, and the central and peripheral analgesia did not appear to be mediated through opioid receptors [46]. Prolonged anti-nociceptive effects have also been demonstrated for clodronate, a non-nitrogenous BP, without inducing any behavioural side effects [47]. Such action has been proposed to explain the efficacy of zoledronate in experimental rat OA models [12]. Increased osteoclastic activity, as found in OA [4,13,17,18], could contribute to neuronal excitation and pain, and, therefore, inhibition of bone resorption and of nociceptor activation through anti-acid, anti-inflammatory actions would be analgesic. This hypothesis maintains an interaction of TLN with the molecular subchondral bone/cartilage cross-talk.

Subchondral bone mineral density decreases as early as one month following ACL transection [48]. In this OA model, extensive remodelling leading to a reduction in subchondral bone plate thickness, bone surface, and trabecular thickness, increased trabecular separation and loss of bone density and anisotropic properties were also reported [6,49-51]. In this study, we found that following TLN treatment a number of those changes were reduced and closer to the values found in normal dogs [6]. More specifically, the architecture of subchondral bone and calcified cartilage was better preserved. The anti-resorptive action of TLN may explain these findings.

The advance movement and duplication of the tidemark contributes to overall thinning of the articular cartilage, thickening of the calcified cartilage while altering the biomechanic of the joint [18,52]. In ACL-deficient dogs, the calcified cartilage undergoes thinning and an advancement of the tidemark later in the OA process [6,48]. However, duplication of the tidemark was previously reported following 12 weeks of BP treatment [21]. Whether or not TLN affects the endochondral ossification remains to be determined as change in the calcification front was not assessed in the present study.

The decrease in cathepsin K activity observed under TLN treatment is well in line with the known mode of action of the drug at reducing osteoclastic activity [9,10]. Given that mechanical loading governs bone architecture and mass, the greater alteration in bone structure found in placebo-treated dogs may also be explained by the lower forces transmitted to the joint as a consequence of pain-related limb disuse. Whereas previous studies using BPs [14,15] or other antiresorptive agents [5-7] focused on the beneficial structural effects in experimental OA models, the present study highlights that the anti-resorptive effect of TLN was associated with an apparent absence of effect on cartilage lesions, but translated to beneficial analgesic and functional consequences. One must, however, take into account that the present study lasted only eight weeks, which is an obvious limiting factor regarding the exploration of the chondroprotective effect of TLN.

A recent analysis using MRI confirmed the common belief that it is likely that changes in the subchondral bone (BML) predominate in relation to OA knee pain [2,53]. Bone marrow lesions are also associated with cartilage lesions [54]. These results support the dynamics of bone/cartilage cross-talk, and the fact that TLN affected the molecular, nociceptive, and biomechanical bone/cartilage interface in the canine ACL model. In the dog ACL model, we have observed similar relationships between MRI structural and functional changes [22,23,55]. Such findings further support the translational nature of results obtained in the ACL dog model to human OA.

## Conclusions

The use of the dog ACL model in association with the complete set of functional methods used in the present study represents a most useful tool for the monitoring of pain and joint function in OA. The present study brings into perspective a possible link between joint structural changes and functional outcomes. The level of joint inflammation is an important co-factor in generating pain-related functional disability. The preservation of bone integrity also likely plays a key role in functional outcome, being required to reduce the disability occurring in ACL-deficient dogs. This is supported by the fact that TLN treatment demonstrated a positive effect on gait disability and joint symptoms, while being associated with a better preservation of calcified cartilage and subchondral bone histomorphometry, as well as reducing the synthesis of catabolic and inflammatory mediators.

## Abbreviations

ACL: anterior/cranial cruciate ligament; ADAMTS: a disintegrin and metalloproteinase domain with thrombospondin motifs; ASIC: acid-sensing ion channel; ATP: adenosine triphosphate; BML: bone marrow lesions; BP: bisphosphonate; DAAI: daily averaged active intensity; DATI: daily averaged active intensity; EDA: electrodermal activity; MIA: mono-iodo-acetate; MMP: matrix metalloproteinase; MRI: magnetic resonance imaging; NOx: nitrites and nitrates; NRS: numerical rating scale; OA: osteoarthritis; P2X: purinergic receptors; PGE_2_: prostaglandin E_2_; PVF: peak vertical force; %BW: percentage of body weight; SC: subcutaneous; SD: standard deviation; TLN: Tiludronate; TRPV1: transient receptor potential vanilloid 1; VAS: visual analog scale.

## Competing interests

The majority of authors declare that they have no competing interests, but Drs T Bertaim and D Thibaud are regular employees of CEVA, who supervised the study for this sponsor.

## Authors' contributions

MM contributed to the study design, carried out the analysis and interpretation of data from the gait analysis, contributed to tissue sampling, performed the statistical analysis and drafted the manuscript. PR carried out the analysis and interpretation of data from the pain and functional evaluation, and participated in the manuscript drafting. JPP and JMP conceived the study, elaborated the design, interpreted the data, and critically revised the manuscript. DL carried out the analysis and interpretation of data from the histomorphometry, and participated in the manuscript drafting. CB carried out the analysis and interpretation of data from the macroscopic and microscopic grading, and participated in the manuscript drafting. JC carried out the analysis and interpretation of data from the macroscopic and microscopic grading, contributed to the management of data from pain and functional evaluation, and participated in the manuscript drafting. DF contributed to the design, analysis and interpretation of data from video-automated behaviour recording, and participated in the manuscript drafting. BL contributed to the surgical process, pain and functional evaluation and critically revised the manuscript. JdC contributed to the statistical analysis and critically revised the manuscript. GB contributed to the statistical analysis, and participated in the manuscript drafting. DG carried out the nitrites and nitrates assays and contributed to the management and interpretation of data from the pain and functional evaluation and revised the manuscript. TB participated in the conception of the study, provided the test article and placebo, participated in the data interpretation and revised the manuscript. DT participated in the conception of the study, provided the test article and placebo, participated in the data interpretation and in the manuscript drafting. ET conceived the study, elaborated the design, managed the experiments, contributed to the pain and functional evaluation, collected the data, sampled tissues, interpreted the data, drafted and revised the manuscript, and is responsible for the integrity of the work as a whole. All authors read and approved the contents of this final version of the manuscript.
